# Healthcare leaders’ and elected politicians’ approach to support-systems and requirements for complying with quality and safety regulation in nursing homes – a case study

**DOI:** 10.1186/s12913-023-09906-6

**Published:** 2023-08-22

**Authors:** Malin Rosell Magerøy, Geir Sverre Braut, Carl Macrae, Siri Wiig

**Affiliations:** 1 SHARE- Centre for Resilience in Healthcare, Faculty of Health Science, University of Stavanger, Stavanger, Norway; 2 Department of Research, Stavanger University Hospital, Stavanger, Norway; 3 Department of Social Science, Western Norway University of Applied Sciences, Sogndal, Norway; 4 Centre for Health, Innovation, Leadership and Learning, Nottingham University Business School, Nottingham, UK

**Keywords:** Leadership, HSE, Quality and patient safety

## Abstract

**Background:**

Healthcare leaders play an important and complex role in managing and handling the dual responsibility of both Health, Safety and Environment (HSE) for workers and quality and patient safety (QPS). There is a need for better understanding of how healthcare leaders and decision makers organize and create support structures to handle these combined responsibilities in practice. The aim of this study was to explore how healthcare leaders and elected politicians organize, control, and follow up the work of HSE and QPS in a Norwegian nursing home context. Moreover, we explore how they interpret, negotiate, and manage the dual responsibility and possible tensions between employee health and safety, and patient safety and quality of service delivery.

**Methods:**

The study was conducted in 2022 as a case study exploring the experience of healthcare leaders and elected politicians in five municipalities responsible for providing nursing homes services in Norway. Elected politicians (18) and healthcare leaders (11) participated in focus group interviews (5) and individual interviews (11). Data were analyzed using inductive thematic analysis.

**Results:**

The analysis identified five main themes explaining how the healthcare leaders and elected politicians organize, control, and follow up the work of HSE and QPS:

1. Establish frameworks and room for maneuver in the work with HSE and QPS.

2. Create good routines and channels for communication and collaboration.

3. Build a culture for a health-promoting work environment and patient safety.

4. Create systems to handle the possible tensions in the dual responsibility between caring for employees and quality and safety in service delivery.

5. Define clear boundaries in responsibility between politics and administration.

**Conclusions:**

The study showed that healthcare leaders and elected politicians who are responsible for ensuring sound systems for quality and safety for both patients and staff, do experience tensions in handling this dual responsibility. They acknowledge the need to create systems and awareness for the responsibility and argue that there is a need to better separate the roles and boundaries between elected politicians and the healthcare administration in the execution of HSE and QPS.

## Background

Healthcare leadership roles are becoming increasingly complex and carry greater responsibility for the performance of employees, the experience of service recipients and the quality of care provision. Healthcare leaders have an important role in managing and handling the dual responsibility of Health, Safety and Environment (HSE) and quality and patient safety (QPS) This includes safety for both patients and staff, having a good work environment, and delivering high quality services [1, 2]. Quality in healthcare is a broad concept that embraces clinical effectiveness, patient safety, and patient experiences [3]. There is a wide array of challenges regarding QPS in the healthcare services, especially those associated with leadership, such as leader competence and efficiency [4]. There is a growing knowledge base for QPS in hospital setting, but there is a need to increase and develop the effort and knowledge in primary care, such as nursing homes and homecare [2, 5].

HSE and QPS are often handled as two separate areas with different legislation that regulates responsibilities in each field [6–8]. However, research as well as practical experience show that it is necessary to look at, and understand, this in a holistic way [1, 9, 10]. QPS should be an integrated part of the HSE work, as safety and well-being for both staff and patients have become a larger part of the leadership agenda. Organizational, cultural, and psychosocial factors are increasingly having important roles in quality, safety, and service delivery, but we do not currently have a clear understanding of leaders` perspectives or strategies for addressing these issues in practice [1, 9, 11]. HSE and QPS are a dual responsibility, but the practical handling of these, and which systems exist and are used, are not well understood. This knowledge gap is addressed in this study in a nursing home context.

### Regulatory context: the current landscape of QPS and HSE in Norwegian municipalities

Responsibility for the public health and care service in Norway is currently divided between the municipalities and the regional health authorities. The municipalities are responsible for the primary health care services, which includes home care services, nursing homes, emergency rooms and general practitioners (GP).

In a Norwegian context, systematic work targeting QPS improvements is a leadership responsibility at all levels. This is regulated in “regulations on management and quality improvement in the health and care service”, the “Health and Care Services Act” and priorities set in the “National action plan for patient safety and quality improvement” [8, 12, 13]. To ensure the health, safety, and welfare of employees, HSE work is regulated in “Regulations on systematic health, environment and safety work in companies”, the internal control regulations [7], and it aims to reduce the risk of adverse events and accidents [14].

**Fig. 1:**
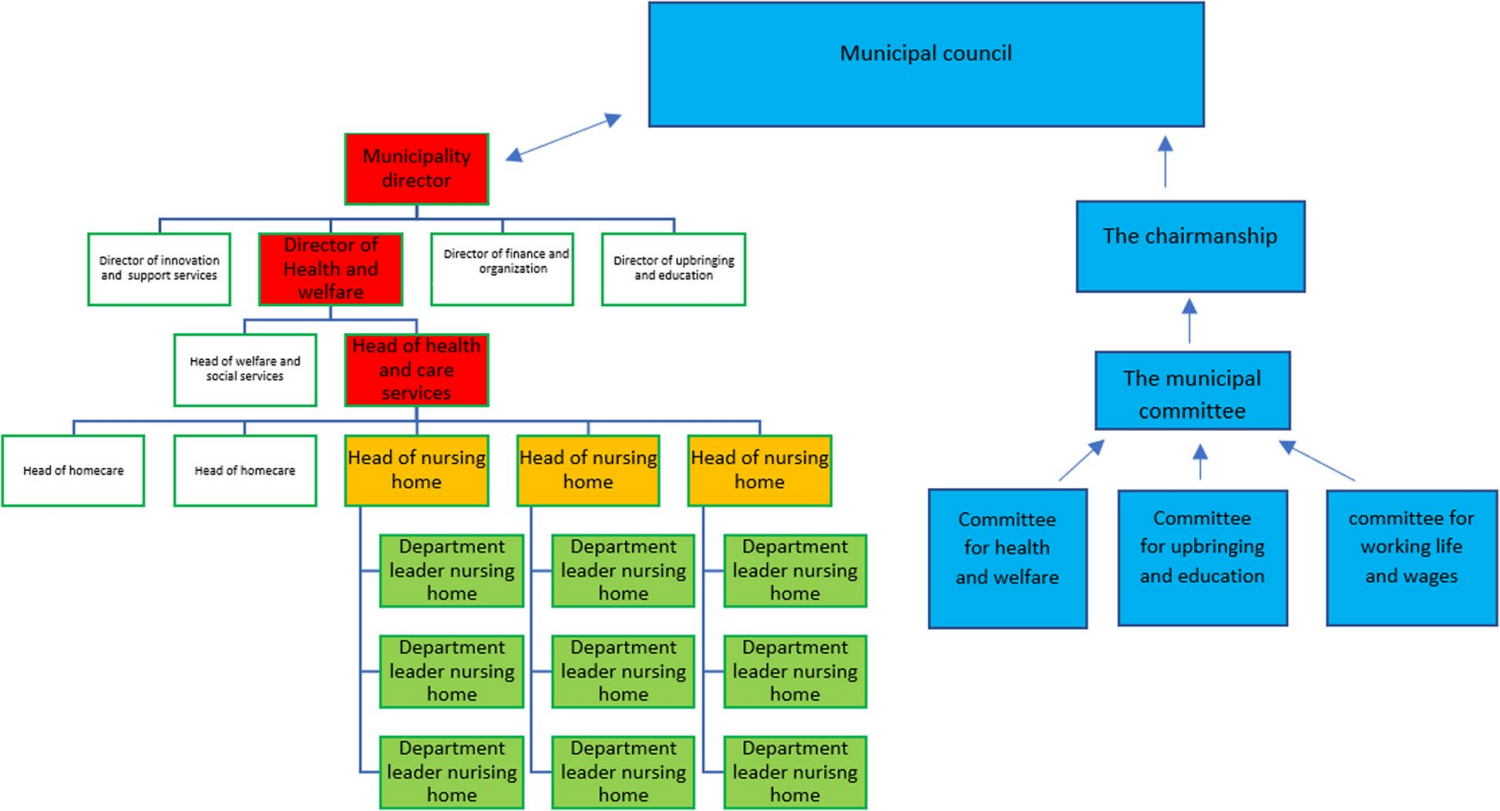
Organization of the municipality according to the chairmanship model. The political steering line is the blue part, while the rest of the organization chart represents the administration. The leadership levels relevant in this study are the red ones

Norwegian municipalities are regulated by the central legal management instrument “Act on Municipalities and County Municipalities” [15], which gives the municipalities a large amount of freedom in how they organize and deliver the services and tasks. The chairmanship model is the most common organization of political governance in Norwegian municipalities. In the chairmanship model, the political committees have the overall control and management of the municipality, with the municipal council as the highest political authority. This makes decisions on behalf of the municipality (see Fig. 1 for organization of the municipality according to the chairmanship model). The council consists of democratically elected representatives and has the overall employer responsibility for all the employees in the municipality. It is therefore important that the elected politicians demand key information about the municipality as an employer, for example about recruiting ability, capacity, and composition of competence in relation to staffing needs, attendance/absence due to illness, figures for turnover including retirement and competence needs in the future [16]. The municipal council is obligated to hire a municipal director as the head of the municipality`s administration. The municipal director is responsible for ensuring that matters submitted to political committees are investigated in a professionally sound manner and that decisions taken in political bodies are implemented, and for the day-to-day exercise of employer responsibility and of all services provided [15].

The “Health and Care Services act” [13], states that the services provided should be justifiable and that the municipalities must work systematically to improve QPS. The “Working Environment Act” states that the work environment should be fully justifiable, injuries and sickness must be prevented, and risk assessment must be conducted [6]. Within this framework, the municipalities are free to organize the work and develop plans and governing documents. This freedom likely enables a wide variety of practical strategies and approaches, but we currently do not have a clear understanding about how the interaction between elected politicians and healthcare leaders affects the quality of services and the safety of patients and staff.

## Aim and research questions

The aim of the study was to explore how healthcare leaders in the municipalities organize, control, and follow up the work of HSE and QPS. We wanted to examine how the leaders and elected politicians interpret, negotiate, and manage the dual responsibility and possible tensions between employee health and safety, and patient safety and quality of service delivery. In addition, we were interested in how the healthcare leaders experienced their own role in this work, and the support available or needed in the process of handling the dual responsibility.

The following research questions guided the study:

1. How do leaders establish support systems and requirements for complying with HSE and QPS regulation in the organization?
2. How do elected politicians get involved in decisions on improving HSE and QPS and how is HSE and QPS reflected in policy documents and plans?
3. What kind of structure, system, tools, and support are available or needed to help leaders at different levels to manage the dual responsibility of HSE and QPS in their organization?

### Understanding the relation between QPS and HSE

One way of considering the dual responsibility of quality and safety for staff and patients is to be guided by the Systems Engineering Initiative for Patient Safety (SEIPS). SEIPS can be regarded as a theoretical perspective that focuses on systems design, and its impact on processes and outcome. Within the SEIPS model there are five components to a work system: person, task, tool/instrument physical environment and organizational conditions [17, 18]. The components interact with and influence each other, and the interaction could result in different outcomes in the domains of performance, safety and health, and quality of working life. The structure within an organization such as a municipality (the work system) affects the safety of the care provided, and the means available to care for the patient (the process) affects patients safety (outcome). The organization in which the health care is provided (work system) affects both the work and the clinical process, which in turn will affect the individual (employee or patient) and organizational outcome [17, 18]. Outcome in the SEIPS model is divided into patient outcomes and employee and organizational outcomes. Changes in the organization will affect the work, clinical processes, and the outcome in a positive or negative way. The SEIPS model aims to explain how all parts of an organization affect and depends on each other and how changes can affect the outcome. In this study the SEIPS model was used as a framework when the research questions and interview guides were formulated and in discussion of the findings.

By using the SEIPS model and investigating the ways elected politicians and leaders in the Norwegian nursing homes context manage the dual responsibility, this article contributes with new knowledge on the current dilemmas, challenges and opportunities in enacting leadership roles and responsibilities for promoting HSE and QPS. This is currently a joint challenge for leaders in healthcare across different healthcare context and countries, and learning from handling these are required for both practical, educational and research purposes.

## Methods

### Design

This study is a part of a single embedded case study. The case is defined in terms of two versions of safety: HSE and QPS in nursing home context and how they are organized, controlled, and followed up and the tension between them from a leadership perspective. The main study consists of three levels of stakeholders and analyses, and this sub-study is exploring the municipality director, director of health and welfare and head of health and care services, and elected politicians` experiences in five Norwegian municipalities (structure showed in Fig. 1).

### Sample and recruitment

The municipalities were recruited through recommendations from the Norwegian Association of Local and Regional Authorities, of which all Norwegian municipalities and county councils are members. The recommended municipalities were contacted with an invitation to participate in the study, together with an attached information letter. The municipalities were selected based on number residents, number of employees in the organization (size), and location (urban/rural). Although this is not a comparative study, having a variation in contextual factors can broaden understanding when exploring the research questions. In this study, leaders are defined as healthcare leaders organizationally placed above head of nursing homes and politicians are defined as democratically elected representatives (see Fig. 1). When the municipality agreed to participate, it meant that (based on the organization) both politicians, municipality director, director of health and welfare, and head of health and care service agreed that they should participate in the data collection (see Table 1 for the characteristics of the participating municipalities). In the rest of this article, the healthcare leaders at all three levels, and the elected politicians, will be referred to as leaders and politicians.

**Table 1:** Characteristics of municipalities and data collection

Municipality	Urban/rural	Citizens	Employees	Individual interviews	Leadership role, education, and years of experience	Focus group
1	urban	42000	3200	3	**1. Role:** Municipality director **Education:** master in economy **Years of leader experience**: 20 **2. Role:** Director of health and welfare. **Education:** administration and organizational science, social economy, and public law. **Years of leader experience:** 20 **3. Role:** Head of health and care service. **Education:** registered nurse, economy, and leadership **Years of leader experience:** 26	1, *N* = 2 **1.Education:** electrician **Years of political experience:** 20 **2. Education:** teacher **Years of political experience:** 7
2	urban	72,000	5300	2	**4. Role**: Municipality director. **Education:** social worker, leadership, and economy. **Years of leader experience:** 27 **5. Role**: Director of health and welfare.**Education:** registered nurse and leadership. **Years of leader experience:** 30	1, *N* = 6 **1.Education:** nurse **Years of political experience:** 16 **2. Education:** real estate agent **Years of political experience:** 10 **3. Education:** civil engineer **Years of political experience:** 11 **4. Education:** high school **Years of political experience:** 33 **5. Education:** appraiser **Years of political experience**: 28 **6. Education:** Economist **Years of political experience:** 32
3	rural	2000	300	2	**6. Role**: Municipality director. **Education:** pedagogy and leadership **Years of leader experience:** 10 **7. Role** Head of health and care service. **Education:** registered nurse, health law, quality development, leadership, and economy. **Years of leader experience:** 3	1, *N* = 3 **1. Education:** social worker **Years of political experience:** 12 **2. Education:** accountant **Years of political experience:** 7 **3. Education:** nurse **Years of political experience:** 15
4	rural	2000	250	2	**8. Role:** Municipality director. **Education:** municipal planner, leadership. **Years of leader experience:** 10 **9**. **Role:** Head of health and care service. **Education**: registered nurse, economy, and leadership. **Years of leader experience:** 18	1, *N* = 4 **1. Education:** teacher **Years of political experience:** 3 **2. Education:** engineer **Years of political experience:** 7 **3. Education:** teacher **Years of political experience:** 12 **4. Education:** social worker **Years of political experience:** 2
5	urban	61,000	5000	2	**10. Role:** Municipality director. **Education:** Pedagogy, religion, Master of Management. **Years of leader experience:** 35 **11. Role:** Head of health and care service. **Education:** economy and administration. **Years of leader experience:** 30	1, *N* = 3 **1. Education:** teacher **Years of political experience:** 8 **2. Education:** electrician **Years of political experience:** 12 **3. Education:** teacher **Years of political experience:** 7

### Data collection

Data collection consisted of 11 individual semi structured interviews with the leaders (*n* = 11), and 5 focus group interviews with politicians (*n* = 18). The leaders had different backgrounds in terms of education and experience, but they all had formal leadership skills and long leadership experience. The politicians had different background in terms of education and experience, and although some of them had been politicians for a long time and had it as a full-time job, others were relatively inexperienced and without a permanent place in the committee they represented. Each focus group consisted of politicians from the same municipality. All interviews, both individual and focus group, were conducted physically in the respective municipality. Due to sickness, 1 individual interview was conducted digitally on Teams. All interviews were conducted from March- June 2022. Semi structured interviews with leaders were conducted by the first author and were based on an interview guide covering the research questions. Topics in the interview guide were inspired by SEIPS [17] and related to *how they organize the work with HSE and QPS, system, tools, and support to work with HSE and QPS, the dual responsibility and interaction with politicians.* The interviews lasted approximately one hour. The focus group interviews covered similar, but slightly different topics than the leaders. Topics covered were *interaction and involvement with the administration, the dual responsibility, overview, and priorities.* One moderator (first author) and one observer (second or fourth author) were responsible for conducting the focus group interviews. The focus groups lasted for approximately 90 min. All interviews were audio recorded and transcribed by the first author. Policy documents and guidelines from all five municipalities were also collected.

### Data analysis

The data material was uploaded in NVivo and analyzed inductively by using thematic analysis. Thematic analysis is suitable to identify, analyze and report patterns or themes within the data, and involves searching across a data set to find repeated patterns of meaning [19]. We used Braun and Clarkes (2006) 6-phase guide to thematic analysis see Table 2.

**Table 2:** Key stages in process of thematic analy

1. Familiarizing yourself with the data	Data was transcribed and red
2. Generating initial codes	Meaning units were extracted, and codes were generated across all data using NVivo
3. Searching for main themes	The different codes were sorted into subthemes and potential themes by using thematic map
4. Reviewing themes	The extracts were re-read, and subthemes were reviewed
5. Defining and naming themes	The essence of each theme was identified, and themes were named
6. Producing the report	The final analysis was completed, and the report were written

The first author (MRM) was responsible for the analysis with input from GSB and SW who red transcripts and discussed theme development throughout the analysis period. The individual interviews and the focus group interviews were coded and categorized separately in 29 sub-themes before they were pooled together based on common characteristics and sorted under five main themes on how top-level leaders in the municipalities organize, control, and follow up the work of HSE and QPS. Factors that influenced this process were also identified. The analysis was discussed in meetings and finally agreed upon by all authors.

## Results

Based on variation in size and organization of the municipalities, the analysis indicated that leaders within each municipality chose different ways of handling the challenges they had, as well as the ways they created an overview and structure of this work. Despite the structural differences in the municipalities the identified themes were recurring and common to all the participants. In the following we present the five themes.

### Establish frameworks and room for maneuver in the work with HSE and QPS

The leaders focused on creating and establishing good frameworks and room for maneuvers to organize, control and follow up the work with HSE and QPS. How the municipality was organized and how they performed their work tasks, the quality of the work conducted, economy, how they created systems, the context they were in, and which tools they had for measurement were factors that influenced the establishment of frameworks and room for maneuver.

#### Size and organization

The fact that the municipalities in this study were different in size and location influenced how they were organized. The leaders in the large municipalities reported a need for a greater degree of structure and independence in the organization, as they become more distant and must to a greater extent rely on good reports and routines to have an overview and control of the work conducted.

Leaders in small municipalities reported that they found it challenging to have the same demands and expectations as the larger ones, as they do not have the expertise and number of employees to handle this, they argued. For leaders in small municipalities this meant that they experienced a wider involvement than leaders in larger municipalities. Especially legal requirements such as requirements for internal control were perceived as a challenge for the smaller municipalities. These leaders called for models or examples of how this could be arranged for small, medium-sized, and large municipalities with their limitation in resources.

*“The requirements are not adapted to small municipalities. It is difficult to have control over all the 2,600 paragraphs that you are supposed to, so we need more support. It would be useful to have a place where you could share knowledge with other leaders in small municipalities”* (leader, municipality 3).

To handle the imposed requirements for services, and to make better use of resources, the small municipalities had established inter-municipal cooperation, and in some place’s collaboration across county borders.

*“We would not have managed without inter-municipal cooperation. The demands for specialized skills are increasing”* (leader, municipality 4).

Although the small municipalities experience some challenges in relation to having the same requirements for quality and safety as large municipalities, they also emphasized some advantages regarding their size. They had a more detailed overview of their status and the lines between leadership levels are short, implying that decisions are made more quickly, and measures initiated. To adapt to changes, be more effective and meet future demands, there have been frequent reorganizations in the municipalities to form larger units with fewer leaders and a more unified competence.

*“We have made many reorganizations the recent years. Now we have arranged the services according to a service model which involves bringing together all the services that deal with the same thing, for example homecare services to one area. This way, there will be fewer leaders, and we really believe in bringing together all the quality and safety of service delivery within an area”* (leader, municipality 2).

One of the participating municipalities was merged from two smaller neighboring municipalities with different systems, culture, and tradition. The leaders spent a lot of time establishing a common system and shared understanding, to provide good working conditions, living conditions and high-quality services in the municipality.

*“For me as a leader it means a new way of working, and a realignment of the geographical zones and redistributing resources so that there is equality across the entire municipality. If you live in old (name of municipality A) or old (name of municipality B), you will get the same offers and services now. This means that we have rebuilt the entire structure and that some employees will have to change workplaces"* (leader, municipality 5).

#### Quality

The concept of quality was difficult for participants to define. Although they were concerned with providing good quality services, they questioned the concept of quality. Both leaders and politicians were concerned that the concept of quality and the subsequent quality work had to be adapted to the recipients of the services. What was perceived as good quality and if this aligned with professional requirements and legislation and agreements, were elements highlighted by the informants.

*“Quality is about the content of the services you receive. That the services are within the standard of the legislation and guidelines, and that there is satisfaction from the user. But the question is always, is the quality good enough? This can be a challenge to discuss and agree on”* (leader, municipality 3).

Quality and competence were seen as areas that are connected and influence each other, as well as the outcome of the service provided. Some of the municipalities had established a quality council to ensure that the services were of good quality and in accordance with the law. All the participating municipalities were concerned with the level of quality and how this could be measured. They used supervision from the authorities to improve practice and create frameworks. The leaders were clear that they did not have good enough routines and systems to measure service quality.

#### Economic situation

Economy was considered a factor that influenced both room for maneuver, quality, and work environment. Financial constraints made it challenging to comply with all guidelines and requirements from the authorities, and there was always a prioritization of which necessary measures to implement. By introducing one thing, they traded off something else, leaders said.

*“We have been in a situation in our municipality where we have made major cuts, and there are still unresolved savings requirements in health care. I feel it in my gut, because we know that there are fewer employees at work, the patients are sicker and in need of complicated care.... how do we ensure that the employees have a safe and good working environment where they feel competent and able to meet the expectations that we have of them? Where is the limit?”* (leader, municipality 2).

Leaders felt it challenging to meet the legal requirements with such a strained economy and experienced that they were measured in terms of efficiency and operating at the lowest possible cost. They conceptualized this as a political responsibility in allocating enough money. The politicians experienced that they had to constantly balance between allocating money and increasing the income of the municipality to achieve budged balance. They wanted state authorities to take a larger share of the cost through increased budgets and frameworks, so that the municipalities could deliver required services.

#### System

The quality system, Compilo, was used by all participating municipalities. Compilo is an electronic system where documents can be stored, and is used to save updated regulations, guidelines, and procedures. Compilo is also used for reporting deviations, such as adverse events, in the participating municipalities. The large municipalities included much of the structures, support, and leading procedures in the system, but the participants still experienced that things were unclear and that they had less control over what was happening compared to the smaller ones. Despite less systems and structures in the small municipalities, participants still felt they were operative, had sufficient overview and control over the challenges and areas for improvement in the municipality.

#### Create good routines and channels for communication and collaboration

The municipalities are facing major challenges in the future in terms of societal expectations, level of care provision, and required knowledge and expertise. To meet these demands leaders focused on establishing good routines for communication and collaboration with both internal and external stakeholders. The leaders said that even if there are more patients and they are sicker and require more demanding care, staffing levels are the same. That placed higher demands on the employees, but also on the municipalities to utilize the expertise in a new and more efficient ways.

*“And then you can say that we try to solve most things with the help of welfare technology and new ways of organizing our services, but ultimately it is about what we communicate and what we are able to provide. I think it is becoming difficult to keep the same level of care, because the patients are getting sicker and sicker. There is no doubt that those who previously died in hospitals now die in nursing homes”* (leader, municipality 5).

#### Competence and Future challenges

How to use the available competence to meet the future demands for the municipalities was a concern for both leaders and politicians. All agreed the need to rethink and restructure, and several of the municipalities were in a process of moving care homes and health care services into the municipality center and required the residents to settle where the services are located. According to the politicians, this was a deliberate policy. They believe that resources will be utilized in a better way, the elderly become more independent as they can walk to the services themselves, and it is assumed that there will be less loneliness if you gather people in one place and create a community.

The municipalities also had to look at how nursing skills could be utilized in the best possible way in the future.

*“When we have a shortage of nurses in the future, then the nurses can only to do what the nurses are trained to do and drop some tasks, for example making breakfast. Nurses don't have to do that. We must refine the nursing tasks and then the healthcare workers must be given greater, expanded competence and responsibility to take on what the nurses do today. But if the healthcare professionals are to do more, then someone else must take over their tasks. We may have to invest in that in the future, that someone can handle everything that has nothing to do with the patient's health, for example setting up breakfast, cleaning, filling linen trolleys and things like that. Now there is such a mix that nurses and healthcare workers do everything, and we don’t use their competence fully”* (leader, municipality 5).

The municipalities are in the process of adopting welfare technology and digital platforms to meet the future needs. The leaders agreed that they had a lot of systems for different areas, but they did not have effective systems that spoke to each other in a holistic way. They were also in need of more human resource and information and communications technology support, since they spent a lot of time extracting reports.

#### Expectations from society

The municipalities must handle societal expectations regarding the design and content of services. The media plays an important role both by informing citizens about what is happening in the municipality and by sharing users' and relatives' stories. Leaders could find media reporting to demanding as it shed light on specific issues (e.g., sick leave, allocation of places in nursing home, and deviations) and set a public narrative and agenda.

The leaders argued that societal expectations have increased, while resources have decreased. They describe that this gap generates a lot of frustration, and especially when the demands are constantly increasing.

*“There is a younger patient with dementia. In the municipality we have care homes, we have nursing homes, and we have home care services. The offer he has received is a place in a nursing home. After all, nursing homes are mostly for the old and the sick, but it is the only offer we can provide, and relatives react to that. It is not a worthy offer for someone who is between 50 and 60 years old, and then we get into the quality issue. What can we offer in addition? And if it is the case that we must constantly think that all offers, all services must be aimed at such criteria..... What is young? Should we have a separate offer for those aged between 50 and 60? 60 and 70 years? We don’t have the resources for that”* (leader, municipality 5).

Leaders were preoccupied with including the patients in the future changes to create trust and understanding. This was done by communication and collaboration.

#### Build a culture for a health-promoting work environment and patient safety

Organizational culture was often invoked to explain challenges and opportunities within both HSE and QPS. To build a health-promoting work environment and improved patient safety, municipalities must work on organizational culture, culture for reporting adverse events, culture for handling sick leave, and full-time culture.

#### Incidents

The municipalities were focusing on adverse events and wanted to increase the number of reported events in Compilo (the reporting and improvement system) and focused on making it known in the municipality and actively used. Leaders were preoccupied with using adverse events to learn and create better systems for HSE and QPS, and they argued they worked continuously to improve reporting culture as they believed they received too few incident reports. The reasons were described as complex, but primarily attributed to staff training, communication, and transparency regarding responses to incidents. They also worked to define what constitutes a deviation, and what is predictable in the work in question.

*“ We encourage them to report. We must know, and we must be open and transparent, I think that`s hugely important. We must have good communication around the challenges that exist if we are to achieve our goals. But then there is this with the reporting culture……”* (leader municipality 2).

The leaders were aware of their responsibilities when serious events occur, and they felt safer and more in control when they set up a system for reporting, receiving, and monitoring incidents, and they instituted these systems in the hope of reducing system failures. By increasing the number of incidents reported, leaders experienced a growing interest from politicians and the media about the reason why the number of adverse events increased.

*“It’s a double thing, because you get front page in the newspaper about all the deviations, and it is perceived negatively. But we encourage the employees to report because we must know, we must have openness and transparency. We need to establish good communication around this subject if we are to achieve our goals. So, therefore we must work with a culture of reporting events and communicating the reason for the increase to politicians and media. It is challenging to get them to understand that when we focus on reporting, the events will increase, but it is not necessarily because we have more incidents, but because we report more”* (leader, municipality 2).

Politicians argued they did not have enough information about the work done and the statistics regarding deviation reports and adverse events and expected to rely on these reports to say something about the quality level of the work done, and how employee safety was affected.

#### Absence

Absenteeism due to sick leave was perceived as high in the municipalities. There were several reasons and implications of this, but according to the participants both leadership, work environment, workload as well as individual factors affected the rate. They agreed that sick- leave affected not only the work environment, but also the quality of the work done and thus patient safety. Building a culture for a health-promoting work-environment was something the leaders found essential. They also believed that responsibility and commitment would reduce sickness absence.

*“We must create the kind of culture where we have a sense of commitment to the organization. I believe that if we manage to make a big organizational commitment, that it is nice to be here, then we will come to work. I also believe that responsibility is important. If you feel that you have responsibility, that you are substantial and important, then the desire to go to work will be quite strong”* (leader, municipality 5).

All leaders and politicians meant that sickness absence affected the work environment as it led to attrition on the other colleagues, as well as a possible culture for low threshold for staying home.

#### Full-time culture

Establishing a culture of working full time and establishing full time positions was on the agenda. Informants believed that full-time culture would reduce sick leave, increase quality, and improve patient safety. As employees working part time will have less knowledge of the patient’s needs, it will provide less continuity and thus unfortunate situations can arise, which would not necessarily occur if professionals worked full-time and know the system and routines.

*“We focus on full-time culture. I think that is essential for several things; The quality of what we do, patient safety, the fact that there are fewer employees, greater continuity, you work with the same, you know the patients, you know what has happened, you are at work more often and you are more involved in taking responsibility. The job will be nicer, and I believe that the HSE work will improve, I believe that the quality of what we deliver will improve and I believe that patient safety will improve”* (leader, municipality 1).

### Create systems to handle the possible tensions in the dual responsibility between caring for employees and quality and safety in service delivery

For several leaders in this study, work with HSE and QPS was difficult to separate. They saw that HSE and QPS often influenced each other and experienced this to be a huge field and an enormous responsibility.

*“It is challenging to meet the expectations on both sides, i.e., employees and patients/relatives. To make this cohere and match”* (leader, municipality 1)

The politicians were concerned with interaction with leaders and administration in this area and pinpointed that there had to be good dialogue between them.

#### Patient safety

Patient safety was seen as important and was defined by the leaders as providing justifiably services, having routines that protect patients’ rights and ensure compliance with law and contractual agreement on how the services are performed. To prevent accidents and adverse events, the leaders in this study argued that competence and routines were important.

*“The right person must provide the right service. There are some medical procedures to be followed, and then you need to have the right skills for it. We must have enough nurses to handle medications. Patient safety also means that we must be sure that the patients will not be exposed to violence or threats from other patients, and of course from staff. And that they have opportunities to live a good life even if they are in a nursing home. That term encompasses so much”* (leader, municipality 5).

The politicians had the same understanding of what patient safety was, but they were also concerned with patients' experience of – and the authority to influence the care provided.

*“It is primarily that the patient feels safe and secure, that they feel looked after and have a dignified and good stay. It must also be the case that the patient is allowed to choose. They should not be a number in a row where everything should go according to fixed routines, but that they should be allowed to have personality and be allowed to do things they enjoy to and experience positively. Patient safety also means that you should get the necessary help, receive medication and that there are competent staff in this institution who see you and do the follow up. Not only in relation to your illness, but also in relation to your personality and the human being that you are”* (politician, municipality1).

#### HSE

When asked about HSE both leaders and politicians were unanimous that it was about health, environment, and safety, (which the letters stand for), but also ensuring security for employees, a good working environment and transparency in the execution of tasks. They were concerned that the concept of quality had to be integrated in the understanding of HSE. To secure quality and maintain the health of both patients and employees, the leaders acknowledged the importance of creating systems and using outcome of the internal control in their quality work. All municipalities had routines for conducting risk-and vulnerability analysis and employee surveys and tried to put them in system.

#### The dual responsibility and different legislation

The dual responsibility of managing HSE and QPS was not a tension that leaders were used to articulating, but they recognized their dual responsibility and experienced this as an ongoing conflict. The leaders recognized that the two domains of safety were regulated by different legislation, and that there is a gap between the Working Environment Act and the main tariff agreement and what they are obliged to do according to the Health and Care Services Act, and the Patient and User Rights Act. They found it challenging to make these fit together.

*“Furnishing in nursing homes is such a dilemma. It is stated in the legislation that the patient is allowed to bring their personal belongings with them when they move in, and this becomes their home. But this poses a risk of falling, risk of infection, who will clean, who will repair, who will look after and so on. The cleaners have their own routines and legislation that they adhere to, so there are immediately conflicts of interest here”* (leader, municipality 1).

#### Priority in the event of conflicting interests

The leaders and politicians were ambiguous and uncertain in their views about how to prioritize in the event of conflict between HSE and QPS. Both employee protection and the service to users are important, and leaders were divided in their response as to what should be prioritized. In most cases they tried to compromise or to resolve the disagreements by communication, however they all found it challenging.

*“This is what makes it so difficult. To read the legislation separately, and then you must put it together and find a way, and everything must be resolved through dialogue. And when it becomes a media issue and a political issue, there is a great desire to win....so it is...., I think it is difficult and I think this is processes that takes long time. I don't think it's easy”* (leader, municipality 1).

For the politicians the choice appeared easier as they are elected by the citizens.

*“We are, after all, elected by the people, so it is the citizens' interests that we must focus on. We are the citizens' ombudsperson”* (politician, municipality 5).

None of the participating municipalities had drawn up guidelines for how to prioritize in the event of conflicting interests between HSE and QPS, nor had they communicated or explored their thoughts on this within the organization. The politicians typically did not have granular insight in this area, but felt that there should not be a contradiction, and that the better they made the municipality as a workplace and ensured rights, opportunities and benefits for the employees, the better results would be achieved in terms of goals, service delivery and patient safety.

### Define clear boundaries in responsibility between politics and administration

The boundaries between politics and administration have been made clear in the Norwegian Municipal Act. The interface between politics and administration is nevertheless complicated, and it can be experienced as difficult to know which tasks and matters the politicians should be involved in and be informed about, and what is the administration's area. This study showed that it is important for the municipalities to define clear boundaries and areas of responsibility so that the work is carried out as efficiently and correctly as possible. The leaders said that the politicians want insight and influence in more than the role description implies, and that they must constantly work to maintain the boundaries. They also argued that the new legislation gives them the opportunity to work more professionally and without distraction from politicians.

*“The politicians are supposed to work at system level. The most important thing they do is that they hire a municipal director. They must trust me because they have given me the authority to follow up the internal control. They shall ask me how I follow up, and if there is an accident, I am the one who is responsible for what has happened”* (leader, municipality 4).

The leaders argued that the politicians sometimes became too operational and closely involved in solving cases. Leaders also experienced that the politicians did not always have enough knowledge when making decisions, and they acknowledged that it was the administrations responsibility to involve politicians more and create good case presentations.

The politicians experienced that the boundaries between politics and administration had placed them at too great a distance, and that they lost control, and received too little insight and ability for participation. It was particularly the politicians in the small municipalities who reported this.

*“We never have these discussions anymore. It is considered in the administration but is not brought up to us as politicians. If we are going to have good discussions and value assessments, we need to gain knowledge, and I feel that this knowledge has been filtered before it reaches us”* (politician, municipality 3).

The politicians in this study enjoyed the position where they could develop society and work to make the conditions of the citizens better. Politicians in the small municipalities were less concerned with fronting their own political party program, and more concerned with finding good solutions for the citizens and the employees of the municipality. They were concerned with how the municipality could become a good place to live, regardless of where you were in life and what your needs were.

## Discussion

**Fig. 2:**
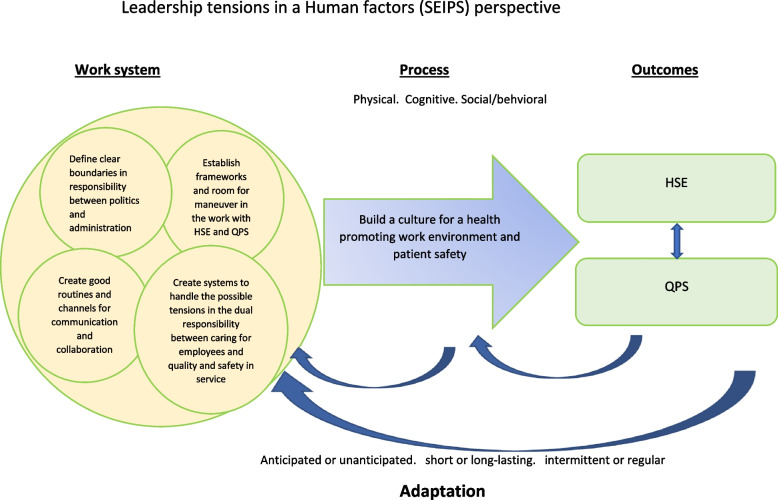
The five themes of how leaders in the municipalities organize, control, and follow up the work of HSE and QPS in a Human factors’ perspective

This study shows the complexity involved for leaders and politicians when enacting the dual responsibility of HSE and QPS (Fig. 2). Several factors influence each other, and the results indicates that both cooperation with others, changes in the organization or how the work is organized internally affect both experience and outcome. As a theoretical framework in this study, we used the SEIPS model to introduce a human factors and system perspective and draw attention to how all components in the work system interact and influence each other [17].

Human factors is a discipline of growing importance for healthcare quality and patient safety [20, 21]. It brings a system orientation which can lead to a shift away from blame -the -person culture to a more holistic approach [22]. The results of this study showed that the municipalities organize, control, and follow up the work with HSE and QPS in various ways. Some of them are common, particularly based on legal requirements and expectations from employees and society, while others are adapted to the individual municipality and their needs. Especially size, economic limitation and available competence influence the daily operations and organization of tasks to handle the dual responsibility of HSE and QPS. This shows how both organizational factors e.g., system, culture, and communication and “external environment”, such as economic- and policy factors outside the organization effects the work system and the outcome [23].

### Size and organization

The requirements for HSE and QPS are the same for all municipalities regardless of size and location, but we found that the rationale for this was questioned, due to lack of competence, capacity, or opportunity to carry out all statutory tasks. Several of the small municipalities found it difficult to fulfill the legal requirements and it may be questioned whether it is sustainable for small municipalities, considering the increasingly complex healthcare service provision, implying high competence level among employees. As argued by Carayon & Perry, it is valuable to listen to such local expertise since all work systems have barriers and facilitators, and these will vary according to context [20]. In our study, the leaders and politicians had the local expertise on their challenges and opportunities, and it was size and location that mainly caused them to experience the situation differently. Our results indicate that there is a need to look at the possibility of adapting legislation and delivery requirements to the context as well as establishing new models to learn from.

To create functional work systems that promotes health, it is important to understand the external environment, such as legislation, requirements, and policy factors [23, 24]. Reform processes in Norway (regionalization and mergers), have resulted in frequent reorganization within and across municipalities. Major change processes can create uncertainty and may have negative effect on the working environment [25], however they may also help ensuring optimal resource use. Healthcare systems must contend with constant changes in conditions and expectations, and must build capacity’s for resilience, to adapt to situation, make changes and learn in order to solve future challenges [20, 26]. Our study showed that there are constant changes in the working conditions in the municipality, both for the organization (work system) and for the healthcare personnel who carry out the task (person) and for the leaders handling the day-to-day decision making. Lack of competence and sick leave were examples in our study which potentially affect employees working conditions (person) and potentially reduce level of service quality. To accommodate these changes, the leaders adapted by organizing work in a different way, using healthcare personnel more efficient and by collaborating with others. Still, this dual responsibility of handling HSE and QPS cause continuous trade-offs for the leaders, while politicians argued the patient’s perspective should be favorable.

All the participating municipalities were concerned with creating systems and structures and acknowledged that this was an area for improvement. The large municipalities had progressed further in this work than the small ones, but all needed to establish framework and room for maneuver. This is consistent with previous research [27] which recognize the need for local autonomy and room for maneuver. In Norway, an expert committee set up by the government has concluded that there should be 15,000 inhabitants as a minimum for municipalities to be able to provide a good range of services in health and care [28]. About 3/4 of the municipalities in Norway have below the recommended number of citizens, and this corresponds to the fact that the leaders in small municipalities in this study found it challenging to deliver all statutory tasks with good quality and sufficient competence and therefore established cooperation across municipalities. The expert committee did not conclude with an upper limit on size of the municipalities, but one may question if a municipality can become too large to operate efficiently and to provide safe and secure services of good quality. We found that to have sufficient overview and control, leaders depended on having system and structure, in addition to a large degree of trust in lower-level leaders and professional advisers. We recommend future studies to investigate this in larger number of municipalities of different size and ways of organizing.

### Quality and reporting

Both politicians and leaders in this study found it difficult to define the concept of quality. Some found the concept too broad and therefore difficult to adopt, while others experienced that there was conceptual room for maneuver that they enjoyed [2]. The room to maneuver related to establishing quality council to ensure quality, review and learn from incident reports. This is in line with previous recommendations [29] where the incident reports is not seen as useful unless they were used for the improvement and understanding of a particular aspect of the organization. Ensuring feedback loops within a work system is fundamental for learning, improvement and adaptation [18]. As a way of increasing QPS and solving lack of competence, a full-time culture with long shifts was used in our case [30]. Some evidence indicates that full-time culture may have a positive effect on work environment efficiency and quality due to a better allocation of work tasks, predictable work schedule, reduced sick leave, and continuity in treatment and care [31]. However, studies also show that long shifts can lead to unintended consequences such as burnout. Full-time culture could reduce the efficiency and effectiveness of the workforce in delivering high quality and safe care [32], which both leaders and politicians should be aware of if moving towards full-time culture. In our study, these negative elements were not noticed, and hence future research should investigate how HSE and QPS may be diversly influenced by changing length of shift. SEIPS 2.0 states that changes in work system (e.g., long shifts), could have a delayed effect on outcomes (e.g., fatigue and turnover) and lead to a higher level of risk and more deviance [23].

### Politics and administration

Changes in the Norwegian legislation were introduced to facilitate a sharper distinction between politics and administration and to clarify tasks, areas of responsibility and control responsibility [15]. A recent study stated that both municipal directors and mayors in Norwegian municipalities experience that the cooperation between politicians and administration is good [33]. Our results differ and indicate that the distinction between politics and administration was perceived differently. The leaders were satisfied with a clear boundary line, and tried to make this visible, while the politicians wanted more information, cooperation, and impact in relation to HSE and QPS. We suggest further longitudinal studies to investigate the implication for handling the dual responsibility and possible consequences for HSE and QPS.

### The dual responsibility

Even though the municipalities have established systems to work with HSE and QPS separately, our results show that the dual responsibility was not a familiar concept for either leaders or politicians. However, tensions were acknowledged, and conflicting interests appeared in daily practice, but there was no prioritization in how to handle these. This echoes previous research claiming that one must look at and understand HSE and QPS in a holistic perspective and that QPS should be an integrated part of the HSE work [1, 9, 10]. This illustrates how the local work system is embedded in a larger socio-organizational context, such as a health care organization (municipality), and that the work systems continuously respond and adapt to changes in the external environment, legislations or regulations, but also to changes in leadership and the way work is organized in the municipality [18]. Our study demonstrates the need for better training and enactment of a holistic perspective and how tradeoffs need to be explicit and handled by both politicians and leaders [18].

### Strengths and limitations of the study

This study is the first to explore leaders` and politicians` experience of the dual responsibility of HSE and QPS, and the support system for organizing, controlling, and following up this work. The strength of this study is that it contributes new insight regarding the challenges faced by leaders and politicians in the municipalities when handling this duality. The experience and challenges explored here are not exhaustive, but they provide insight that may be transferrable to other similar contexts [34]. The study has some limitations. The participating municipalities were recruited through recommendation from the Norwegian Association of Local and Regional Authorities, with some risk of selection bias or unintended pressure to participate in the study. All participants were informed both verbally and in writing that they could withdraw from the study at any time. When using a semi structured interview guide, it is possible that participants may be prompted to answer in a certain way [35]. To minimize that risk, we informed participants that we were interested in their experiences, and that there was no right or wrong answer. Focus group interviews may introduce bias related to group dynamics, for example that participants have different positions, some with more power and higher rank then others, and different degrees of introversion and extroversion [36]. During the focus group interviews this was taken into consideration by having both a moderator and a secretary present to monitor group dynamics, and by attentively giving quieter participants opportunities to speak.

## Conclusion

In this study we have explored how leaders and politicians in five Norwegian municipalities organize, control, and follow up HSE and QPS, and how they experience and manage the dual responsibility of HSE and QPS. The study showed that both internal (organization, system, competence) and external (size and location, legislation, economic situation) factors influence how they experience the dual responsibility for HSE and QPS, and work to handle it. In particular, the size of the municipality influenced the experience, and what systems and structures they have established to implement statutory requirements with good quality in their own context. The study showed that leaders and politicians experience tensions in handling this dual responsibility. They acknowledge the need to create systems and awareness for the responsibilities and argue that there is a need to better separate the roles and boundaries between politicians and the administration in relation to HSE and QPS. This study confirms that a change in one of the system components, e.g., reorganization that changes the size of the unit (the work system), may affect how the work is carried out in a positive or negative way due to subsequent changes in the economy, available expertise, etc. (outcome). As depicted in Fig. 2 we have theorized leadership tensions from a human factors perspective to develop a better understanding of how leaders and politicians maneuver to handle the dual responsibility of HSE and QPS particularly in relation to the organization and context of Norwegian municipalities. Future research and practice may benefit from such an approach to promote a more holistic understanding of the input, process and outcomes related to leadership dualities in HSE and QPS.

## Data Availability

The data material is available on request from the corresponding author.

## Abbreviations

HSE: Health, Safety and Environment
QPS: Quality and patient safety
SEIPS: Systems Engineering Initiative for Patient Safety
